# Endogenous Retroviral Sequences Behave as Putative Enhancers Controlling Gene Expression through HP1-Regulated Long-Range Chromatin Interactions

**DOI:** 10.3390/cells11152392

**Published:** 2022-08-03

**Authors:** Sébastien Calvet, Séphora Sallis, Nehmé Saksouk, Cosette Rebouissou, Catherine Teyssier, Annick Lesne, Florence Cammas, Thierry Forné

**Affiliations:** 1 Institut de Recherche en Cancérologie de Montpellier (IRCM), University of Montpellier, Inserm U1194, ICM, CNRS, F-34298 Montpellier, France; sebastien.calvet@etu.univ-lyon1.fr (S.C.); nehme78@gmail.com (N.S.); catherine.teyssier@inserm.fr (C.T.); 2 Institut de Génétique Moléculaire de Montpellier (IGMM), University of Montpellier, CNRS, F-34293 Montpellier, France; sephora.sallis@hotmail.fr (S.S.); cosette.rebouissou@igmm.cnrs.fr (C.R.); annick.lesne@igmm.cnrs.fr (A.L.); 3 Laboratoire de Physique Théorique de la Matière Condensée, LPTMC, Sorbonne Université, CNRS, F-75252 Paris, France

**Keywords:** endogenous retroviruses, HP1, chromatin organization, *Trim24*

## Abstract

About half of the mammalian genome is constituted of repeated elements, among which endogenous retroviruses (ERVs) are known to influence gene expression and cancer development. The HP1 (Heterochromatin Protein 1) proteins are known to be essential for heterochromatin establishment and function and its loss in hepatocytes leads to the reactivation of specific ERVs and to liver tumorigenesis. Here, by studying two ERVs located upstream of genes upregulated upon loss of HP1, *Mbd1* and *Trim24*, we show that these HP1-dependent ERVs behave as either alternative promoters or as putative enhancers forming a loop with promoters of endogenous genes depending on the genomic context and HP1 expression level. These ERVs are characterised by a specific HP1-independent enrichment in heterochromatin-associated marks H3K9me3 and H4K20me3 as well as in the enhancer-specific mark H3K4me1, a combination that might represent a bookmark of putative ERV-derived enhancers. These ERVs are further enriched in a HP1-dependent manner in H3K27me3, suggesting a critical role of this mark together with HP1 in the silencing of the ERVs, as well as for the repression of the associated genes. Altogether, these results lead to the identification of a new regulatory hub involving the HP1-dependent formation of a physical loop between specific ERVs and endogenous genes.

## 1. Introduction

Endogenous retroviruses (ERVs) are remnants of ancient retroviral integrations into the germline. These elements are abundant in mammals, occupying approximately 8% of the mouse genome and 10% of the human genome [1,2]. ERVs were originally subdivided into three distinct classes (I, II, and III) based on the similarity of their reverse transcriptase genes, or on their relationship to different exogenous retroviruses [3]. They constitute a threat for genome stability because they can integrate anywhere in the genome and their expression may interfere with the expression of the host genome. Most organisms have developed efficient silencing mechanisms involving heterochromatin formation that render ERVs unable to be transcribed and/or retro-transposed [4]. However, cellular transcription factors frequently bind long terminal repeats (LTRs) sequences and some ERVs have been co-opted by their host genome, providing an abundant source of regulatory elements that contribute to species-specific transcription-regulatory networks [5,6]. Despite accumulating reports showing that ERV have been co-opted by various host genomes, the mechanisms by which ERV sequences escape silencing to control endogenous gene expression remain poorly understood. We recently showed that the Heterochromatin Protein 1 (HP1) proteins are implicated in the silencing of some specific ERVs within adult livers [7]. The HP1 proteins are evolutionarily conserved proteins with three isoforms in mammals (HP1α, HP1β and HP1γ) that are all enriched in constitutive heterochromatin, although to different extents, and are essential for most functions of this nuclear compartment [8,9]. We also showed that the inactivation of all three HP1 isoforms, specifically in mouse hepatocytes (HP1-TKO), leads to liver tumours, and provided evidence suggesting that this could be explained, at least partially, by the reactivation of specific ERVs and the concomitant altered expression of genes in their vicinity [7]. Because ERV reactivation is believed to play a critical role in cancer, it is of utmost importance to understand the mechanisms regulating ERV expression and their impact on the expression of the genome [10]. In this respect, our HP1-TKO animal model constitutes a precious tool to address these issues.

Here, we explore in detail the link between ERVs, the expression of endogenous genes and HP1. To this end, we investigate specific ERVs of the VL30-LTR class at two loci that have previously been shown to be associated with liver tumorigenesis [11,12] and that are both upregulated in HP1-TKO mouse livers [7]: the *Mbd1* (*Methyl-CpG-binding domain protein 1*) and *Trim24* (*Tripartite motif-containing 24*) gene loci. Using a combination of high-throughput gene expression analysis (RNA-seq) and quantitative Chromatin Conformation Capture (3C-qPCR) [13,14], we show that, depending of the genomic context, LTRs of the VL30-class ERVs are able to act either as alternative promoters or as putative enhancers, forming chromatin loops with promoters of endogenous genes, and that HP1 can control both activities of these elements. We further show that HP1-dependent ERVs are characterised by a specific epigenetic landscape that is partially remodelled upon the loss of HP1 and which could thus constitute a signature to identify HP1-dependent ERVs with potentially deleterious effects on gene expression and liver homeostasis.

## 2. Materials and Methods

### 2.1. Mouse Strains

Mice carrying the triple deletion of HP1 proteins in their liver were obtained as previously described [7]. In brief, the gene encoding HP1α was inactivated constitutively in all tissues. Genes coding for HP1β and HP1γ were surrounded by LoxP sites (the floxed alleles produced intact proteins) and a CRE recombinase under the control of the *Albumin* gene promoter (*Alb*-CRE) was used to inactivate them only in hepatocytes. All mice were age-matched and whenever possible were littermates. To obtain control and HP1-TKO littermates, females of the following genotype [heterozygous HP1α+/−; HP1βflox homozygous (f/f); HP1γflox homozygous (f/f); Tg0/0] were crossed with males [HP1α (+/−); HP1β f/f; HP1γ f/f; *Alb*-Cre heterozygous (Tg *Alb*-Cre/0)]. One-eighth of the mice were thus HP1-TKO (homozygous deletions of all three genes coding for the HP1 [HP1α−/−; HP1β f/f; HP1γ f/f; Tg *Alb*-Cre/0)] called HP1-TKO for simplicity and 1/8 are controls ([HP1α+/+; HP1β f/f; HP1γ f/f; Tg0/0] or ([HP1α+/−; HP1β f/f; HP1γ f/f; Tg0/0) called Ctl. Mice were genotyped as previously described [7]. The rates of HP1α, HP1β and HP1γ gene deletions were determined on the same genomic DNA samples used for 3C-qPCR experiments (cf. below). Each gene was quantified by qPCR (primer sequences are provided in Supplementary Table S1) and the percentage of remaining unrecombined genes in the HP1-TKO mouse liver was calculated relative to their control littermate mice. No HP1α-encoding gene was detected (Supplementary Figure S1) (constitutive KO). The fraction of remaining unrecombined genes encoding HP1β and HP1γ had a mean of 56 ± 10% and 66 ± 20%, respectively (hepatocyte-specific conditional KO) (Supplementary Figure S1). This result is in good agreement with the histological composition of the 7-week-old mouse liver, about 60% of which is composed of hepatocytes.

All experimental designs and procedures are in agreement with the guidelines of the animal ethics committee of the French “Ministère de l’Agriculture” (European directive 2010/63/EU).

### 2.2. Reverse Transcription (RT) and 5′ RACE

Total RNA from livers of 7-week-old mice was extracted using Trizol (Ambion, Austin, TX, USA). Samples of HP1-TKO used in our experiments are numbered 207, 248 and 316, while control samples are numbered 208, 251 and 315 (note that samples 207, 208, 315 and 316 were also used in the 3C assays, see below). Reverse transcription (RT) reactions were performed with 1.5 μg RNA using random hexamer primers and Superscript III from Invitrogen (ThermoScientific, Waltham, MA, USA) following supplier recommendations. All RNA levels determined in RT-qPCR were normalised relative to *Gapdh* mRNA levels.

For RT-qPCR analyses of the RTLV6-18 and RTLV6-86 ERV sequences (upstream of the *Trim24* promoter), specific primer pairs were designed and the number of copies amplified by each primer pair was determined on serial dilutions of genomic DNA (standard curves) in comparison with a control primer pair (“1 copy CTL”) targeting exon 1 of the Krüppel-like factor 4 (*Klf4*) gene, which amplifies exactly one copy in the mouse genome. For the RTLV6-18 primer pair (“1 copy”), we obtained an intercept value identical to that of the *Klf4* primer pair (25.6 vs. 25.1, respectively), indicating that this primer pair is amplifying a unique sequence. In contrast, the RTLV6-86 primer pair (“100 copies”) displayed a difference of about 6 Ct (19.8 vs. 25.1), revealing that the amplified sequence is about 100 times more abundant in the genome.

Rapid Amplification of 5′ Complementary DNA Ends (5′RACE) was performed on total capped RNAs from 7-week-old HP1-TKO mouse liver according to the manufacturer’s instructions (GeneRacer^TM^ Kit from Invitrogen ref. L1502, Carlsbad, CA, USA). Random hexamer primers (Thermo Fisher Scientific, ref. SO142, Waltham, MA, USA) were used for RT, and PCR reactions were performed using the *Mbd1* gene primer and the GeneRacer^TM^ 5′ primer, the sequences of which are provided in Supplementary Table S1.

### 2.3. Chromatin Immunoprecipitation (ChIP-qPCR) Assays

A total of 400 mg of flash-frozen liver biopsies were sliced into around 2 mm pieces with scalpel blades, fixed immediately in 10% formaldehyde solution for 10 min at room temperature, quenched with 0.125 M glycine for 5 min at room temperature and washed twice with cold PBS. The pellet was then homogenised with a Dounce in PBS, filtrated on 100 µm nylon mesh and the cells recovered by centrifugation at 1000 rpm for 10 min at 4 °C. The pellet was resuspended in 600 µL of lysis buffer (50 mM HEPES pH 7.5, 140 mM NaCl, 1 mM EDTA pH 8, 1% Triton X-100, 0.1% sodium deoxycholate) and sonicated for 10 min (30 s On, 30 s Off) using the Bioruptor^®^ Pico (Diagenode, Seraing, Belgium). Debris were removed after centrifugation at 8000 rpm for 10 min at 4 °C. A total of 20–25 µg of chromatin was immunoprecipitated with each one of the antibodies using BSA and salmon-sperm-coated protein G dynabeads^®^ (Invitrogen) overnight at 4 °C. The immunoprecipitations were washed once with low-salt buffer (0.1% SDS, 1% Triton X-100, 2 mM EDTA, 20 mM Tris-HCl pH 8, 150 mM NaCl), once with high-salt buffer (0.1% SDS, 1% Triton X-100, 2 mM EDTA, 20 mM Tris-HCl pH 8, 500 mM NaCl) and once with LiCl buffer (0.25 M LiCl, 1% NP-40, 1 mM EDTA, 10 mM Tris-HCl pH 8). ChIP products were de-crosslinked, purified by phenol/chloroform extraction and ethanol precipitation and resuspended in 100 µL TE buffer. A total of 2 µL was used per qPCR reaction. Relative primer efficiencies were determined on serial dilutions of genomic DNA and taken into account, so that for each epigenetic mark, quantifications can be compared between different genomic sites. The antibodies used for these experiments were pAb H3K4me1 (# 53265; Cell Signaling, Danvers, MA, USA), pAb H4K20me3 (#ab9053; Abcam, Cambridge, UK); pAb H3K27me3 (#DAM1514011 Milipore, Merck, Paris, France), pAb H3K9me3 (#ab5819; Abcam); mAb H3K9ac (#9649; Cell Signaling), pAb H3K4me3 (#ab8580; Abcam), and pAb H3K27ac (#4353; Cell Signaling).

### 2.4. Chromatin Conformation Capture (3C) Assays

The 3C samples were prepared from livers of four HP1-TKO (201, 207, 209 and 316) and four controls (202, 208, 210 and 315) 7-week-old mice. Chromatin Conformation Capture (3C) assays were performed as described in [15,16] with some adaptations as described below.

Nuclei preparations were obtained as previously described [17]. Briefly, livers of HP1-TKO and control mice were dissected from 7-week-old animals, cut into pieces and placed into a Potter homogenisator containing 20 mL of Homogenizer Buffer (HB) (2.1 M Sucrose, 10 mM Hepes buffer pH 7.6, 2 mM EDTA pH 8.0, 15 mM KCl, 10% *v*/*v* glycerol, 0.15 mM spermine, 0.5 mM spermidine, 0.5 mM DTT, 0.5 mM PMSF, 7 µg/mL aprotinine). Homogenisation was performed on ice with four strokes. After a filtration step on gauze, the solution was loaded onto a 15 mL cushion of HB and centrifuged for 40 min at 100,000× *g* and 4 °C into a SW40 ultracentrifugation tube. Aggregates that were floating were removed and the supernatant was carefully put into the sink. The pellet was suspended in 2 mL of wash buffer (10 mM Tris-HCl pH 7.4, 15 mM NaCl, 60 mM KCl, 0.15 mM spermine, 0.5 mM spermidine) and transferred into a 12 mL Greiner tube (Greiner Bio-One GmbH, Kremsmünster, Austria) for centrifugation during 5 min at 5000 rpm and 4 °C. Before this centrifugation, a few drops were taken and the nuclei were counted on a Thoma’s cell. The pellet was finally suspended in an appropriate volume of glycerol buffer (40% *v*/*v* glycerol, 50 mM Tris-HCl pH 8.3, 5 mM MgCl_2_, 0.1 mM EDTA pH 8.0) to have 5 million nuclei in 100 μL of solution. These 100 μL aliquots were frozen in liquid nitrogen and kept at −80 °C.

A 100 μL aliquot containing 5 million nuclei was completed to 700 μL with a 3C buffer (50 mM Tris-HCl pH 8.0; 10 mM MgCl_2_; 50 mM NaCl; 1 mM DTT). Nuclei were carefully suspended with the pipette and left for 5 min at room temperature. A total of 19.7 μL of formaldehyde (final concentration 1%) was added and the tube was maintained at room temperature for precisely 10 min. A total of 80 μL of 1.25 M glycine (125 mM final) was added to neutralise the formaldehyde and the tube was left at room temperature for precisely 2 min. The reaction was then placed on ice for at least 5 min and centrifuged at room temperature for 3 min at 2300× *g*. The supernatant was removed and the pellet was carefully suspended with the pipette by adding 1 mL of 3C buffer. The tube was then centrifuged for 3 min at 2300× *g* at room temperature and the supernatant was removed.

The pellet was then taken into 0.1 mL of 3C buffer and transferred to a Safelock tube. A total of 1 μL of 20% (*w*/*v*) SDS (0.2% final) was added and the tube was incubated at 37 °C for 60 min in a ThermoMixer C^®^ (Eppendorf, Hamburg, Germany) while shaking at 350 rpm. A total of 16.8 μL of 10% (*v*/*v*) Triton X-100 diluted in ligation buffer (40 mM Tris-HCl pH 7.8; 10 mM MgCl_2_; 10 mM DTT; 5 mM ATP) was added. The tubes were incubated at 37 °C for 60 min while shaking at 350 rpm. A total of 10 μL of the sample was saved (“undigested control”) and stored at −20 °C until use for the determination of digestion efficiencies (see below).

A total of 450 U of the HindIII restriction enzyme was added to the remaining sample (3 μL of HindIII at 50 U/µL was added three times by intervals of 2 h) and the sample was incubated for 24 h at 37 °C while shaking gently at 350 rpm (ThermoMixer C^®^). A total of 10 μL of the sample was saved (“digested control”) and stored at −20 °C until use for the determination of digestion efficiencies (see below).

A total of 12 μL of 20% (*v*/*v*) SDS (1.6% final) was added to the remaining sample, and the tube was incubated for 30 min at 37 °C while shaking gently at 350 rpm (ThermoMixer C^®^). The reaction was then transferred with caution into a 12 mL tube (Greiner) and 3.28 mL of ligation buffer was added, along with 390 μL of 10% (*v*/*v*) Triton X-100 diluted in ligation buffer. The tube was incubated for 2 h at 37 °C while shaking gently at 450 rpm (ThermoMixer C^®^), centrifuged for 1 min at 7500 rpm at 4 °C and placed on ice. A total of 3.27 mL of the supernatant was removed to leave 500 μL in the tube. A total of 6.5 μL of ligase HC (30 U/μL) was then added along with 3 μL of 100 mM ATP. The samples were incubated overnight at 16 °C while shaking gently at 350 rpm (ThermoMixer C^®^).

A total of 2 mL of 2× PK buffer (20 mM Tris-HCl pH 8.0, 10 mM EDTA pH 8.0, 1% *w*/*v* SDS) and 1.5 mL of water were added to the tube, as well as 5 μL of 20 mg/mL Proteinase K (100 μg final). The tube was incubated for 1 h at 50 °C and then 4 h at 65 °C to de-crosslink the sample. The genomic DNA was extracted from this reaction by classical phenol/chloroform extraction and ethanol precipitation, and it was suspended in 50 μL of water. A total of 250 μL of 2× StyI restriction buffer (commercial 10× buffer diluted at 2× with water), and 190 μL of water were added and the reaction was placed into a 1.5 mL tube. A total of 5 μL of 1 mg/mL RNase A (5 μg final) and 10 μL of 10 U/μL (100 U final) of Sty I enzyme (Eco130I, Fermentas, Burlington, ON, Canada) were added and the reaction was incubated for 2 h 30 at 37 °C. Genomic DNA was then extracted by phenol/chloroform extraction and ethanol precipitation and suspended in water at a concentration of ~25 ng/µL.

### 2.5. Determination of Digestion Efficiencies of 3C Assays

A total of 500 μL of PK buffer (5 mM EDTA pH 8.0; 10 mM Tris–HCl pH 8.0; 0.5% SDS), as well as 1 μL of 20 mg/mL Proteinase K (20 μg final), was added to the “undigested” and “digested” controls (see above) and the tubes were incubated overnight at 65 °C. A total of 1 μL of 1 mg/mL RNase A (1 μg final) was added to each tube and they were incubated for 2 h at 37 °C. Genomic DNA was extracted by phenol–chloroform–isoamyl alcohol 25:24:1 (*v*/*v*) extraction followed by ethanol precipitation and each pellet was suspended in 500 μL of 1× StyI restriction buffer (commercial 10× buffer diluted to 1× with water). A total of 5 μL of 10 U/μL StyI enzyme was added and the tubes were incubated for 2 h 30 at 37 °C. Phenol/chloroform extractions and ethanol precipitations were performed and the genomic DNA was suspended in 60 μL of water.

### 2.6. Control of Primer Efficiency

A control template containing all ligation products in equimolar amounts was used to optimise real-time quantitative PCR (qPCR) reactions, determine the efficiency of each qPCR primer pair and, for each primer pair, to establish the minimal amount of ligation product that can be quantified in a reliable manner. To obtain this control template, a set of minimally overlapping BAC clones (RP23-211E15 and RP23-9J17) was mixed in equimolar amounts and cut with HindIII before being re-ligated by the T4 DNA ligase. A secondary digestion with the StyI restriction enzyme was performed. Serial dilutions of the control template were used to obtain standard curves for each qPCR primer pair used in 3C-qPCR experiments. To mimic 3C sample conditions, the total DNA concentration of these dilutions was adjusted to ~25 ng/μL using a solution containing mouse genomic DNA at a known concentration.

### 2.7. Real-Time Quantitative PCR

The original 3C samples were adjusted with H_2_O to approximately 25 ng/μL +/− 10% and, for each adjusted 3C sample, real-time qPCR quantifications were performed to obtain the Ct of each ligation product on 1 μL (containing ~25 ng of DNA). Reaction conditions were as follows (10 μL final reaction volume): 1 μL of sample, 1 μL of primer pair (5 μM each), 1 μL of qPCR mix, and 7 μL of H_2_O. The 3C products were quantified in triplicate using a LightCycler 480 II (Roche, Basel, Switzerland) (10 min at 95 °C followed by 45 cycles of 10 s at 95 °C/8 s at 69 °C/14 s at 72 °C) using the Hot-Start Taq Platinum Polymerase (Life Technologies, Carlsbad, CA, USA) and the following qPCR mix [18]: 0.24% W1 (polyoxyethylene ether W1); 500 µg/mL BSA; 300 µM dNTP; 50 mM KCl; 30 mM MgCl_2_; 1/3000 SYBR Green (10,000× in DMSO, LONZA, ref. 50513); 16.24% glycerol; and 400 mM 2-amino-2-methyl-1,3-propanediol buffer mixed to pH 8.3 using HCl. Primer sequences are provided in the Supplementary Table S1.

Quantification values obtained were corrected for potential differences in primer efficiencies and normalised to the “Basal Interaction Level” as previously described [15], yielding the relative crosslinking frequencies presented in the Figures.

### 2.8. Luciferase Enhancer-Reporter Assays

A DNA fragment corresponding to the full *Trim24* RLTR6-86 sequence was obtained by PCR amplification on genomic DNA with a specific primer pair (see Supplementary Table S1) that was designed just upstream and downstream of the *Trim24* RLTR6-86 element in non-repeated sequences. A SmaI restriction site was added at the 5′-end of the forward primer and restriction sites for BglII and KpnI were added at the 5′-end of the reverse primer. The resulting 630 bp fragment was cloned into the “pGL3promoter” vector (firefly luciferase under the control of the SV40 promoter; Promega, Madison, WI, USA) using SmaI and BglII or SmaI and KpnI restriction sites for the forward and reverse constructs, respectively (according to *Trim24* gene orientation in the mouse genome). All constructs were checked by sequencing. A total of 300,000 primary Bipotential Mouse Embryonic Liver (BMEL) cells, derived from E14.5 embryos expressing CTL or not (HP1-TKO) [7], were placed in 96-well plates and transfected the next day with the reporter constructs, together with the Renilla luciferase (Rluc) control reporter vector pRL-CMV (Promega) using the Lipofectamine 2000 transfection reagent (Invitrogen) according to the supplier’s protocol. A total of 48 h after transfection, luciferase activity was determined with a dual luciferase reporter assay system (Promega) and luminescence was measured using a microplate luminometer Centro (Berthold Technologies, Bad Wildbad, Germany). Transfection data were normalised to the Renilla activity and expressed as relative luciferase activity.

## 3. Results

### 3.1. Endogenous and ERV-Derived Upstream Mbd1 Promoters Are Both Controlled by HP1 Proteins

**Figure 1:**
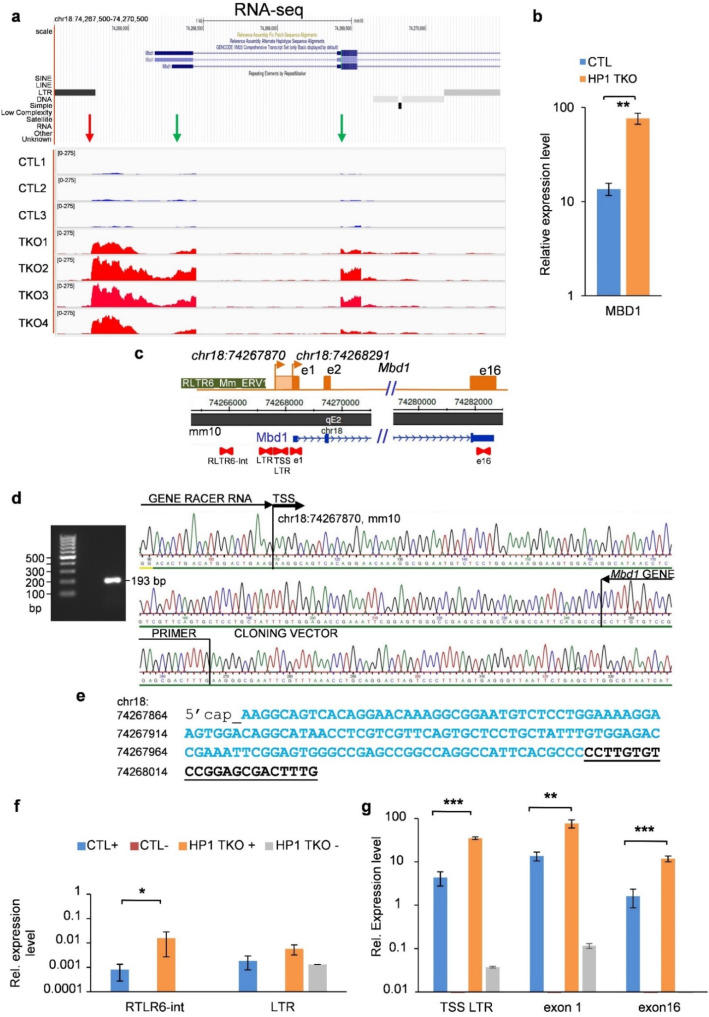
Endogenous and ERV-derived upstream promoters of *Mbd1* are controlled by HP1 proteins. (**a**) Genome Browser snapshot of RNA-seq data at the *Mbd1* locus. *Mbd1* mRNA levels were found to be highly increased in HP1-TKO compared to control livers (green arrows), as well as in one intergenic LTR sequence located 517 bp upstream of the Transcriptional Start Site of the endogenous *Mbd1* gene promoter (red arrow on the left). (**b**) RT-qPCRs were performed on total RNA from three HP1-TKO (201, 248 and 316) and three control (202, 251 and 315) mouse liver samples. Primers targeting *Mbd1* exon 1 were used and RNA levels were normalised relative to *Gapdh* mRNA levels. We observe a significant difference in expression level between the two conditions. (**c**) Map of the mouse *Mbd1* locus (chr18:74,089,445–74,522,642, mm10 assembly). Primer pairs used for RT-qPCR are represented by red arrows. (**d**) A 5′RACE experiment was performed on total capped RNA from TKO mouse liver. Random hexamer primers were used for RT and PCR reactions were performed using the Mbd1_gene primer and the GeneRacer^TM^ 5′ Primer (Supplementary Table S1) located in the GeneRacer RNA indicated in the figure (right panel). The ethidium bromide staining of 1% agarose gel shows the PCR product obtained before cloning and sequencing (left panel). The TSS of the endogenous *Mbd1* promoter is located at position chr18:74,268,291 of the mouse mm10 assembly in C57BL-6 liver and the upstream TSS is located at position chr18:74,267,870. (**e**) The genomic sequence of the transcript issued from the upstream TSS is given in blue and the Mbd1_gene primer sequence used for PCR amplification is underlined in black. (**f**) Reverse Transcriptions (RT) were performed on total liver RNA from control (CTL) and HP1-TKO mice using primer pairs that target single-copy sequences of the RLTR6-int or RLTR6B elements located upstream of the endogenous *Mbd1* promoter (+). Relative expression levels are depicted in bar graphs after normalizing for RNA loading using *Gapdh* mRNA levels. Control reactions without RT were also performed (-) and they remained below detection limits in control mice (CTL-), while they remained very low in HP1-TKO mice (HP1-TKO-) (grey bars). (**g**) Relative expression levels of *Mbd1* upstream transcript (TSS LTR), and exon 1 or exon 16 containing mRNAs, were measured as described above. Error bars represent s.e.m. of 3 biological replicates. *p*-value < 0.01 (***), *p*-value < 0.02 (**), and *p*-value < 0.05 (*) (Student’s *t*-test).

We previously showed that a subset of ERV elements are reactivated upon the depletion of all HP1 isoforms within mouse liver (HP1-TKO) and that this reactivation correlates with the upregulation of endogenous genes in their vicinity [7]. To explore in more detail the interplay between ERVs and HP1 proteins in the regulation of gene expression, we first investigated the mouse *Mbd1* gene, which was previously shown to be upregulated upon the depletion of HP1 proteins [7]. The upregulation of the *Mbd1* gene in HP1-TKO compared to control livers of 8-week-old mice, first observed by RNA-seq (Figure 1a), was confirmed by RT-qPCR (Figure 1b). This coincided with the transcriptional upregulation of sequences immediately downstream of the intergenic ERV-LTR element located 517 bp upstream of the *Mbd1* promoter (red arrow in Figure 1a and RLTR6_Mm_ERV1 in Figure 1c). This suggests that this ERV-LTR sequence behaves as an HP1-dependent alternative promoter.

According to Registry V3 of the ENCODE screen for cCREs (candidate cis-regulatory elements) [19], the endogenous *Mbd1* promoter (EM10E1070191) spans positions chr18:74,267,966 to 74,268,315 (mouse mm10 assembly) in the C57BL-6 liver and the endogenous TSS map at position chr18:74,268,291 (Figure 1c). To characterise the TSS of the upstream promoter, we performed a 5′RACE experiment on total RNA from TKO mouse liver and found that it is located at position chr18:74,267,870, i.e., 421 bp upstream of the endogenous *Mbd1* TSS and only 96 bp downstream of the RLTR6_Mm_ERV1 (Figure 1d,e).

We then designed a strategy to more accurately measure the RNA levels within this ERV and its downstream sequences. In control liver, primer pairs located within the ERV (RTLR6-int) or overlapping the TSS of the upstream promoter (LTR) display extremely low expression levels (0.0008 ± 0.0005 and 0.002 ± 0.001, respectively) (Figure 1f, blue bars). In contrast, a primer pair located just downstream of this last position (TSS LTR) displays a much higher expression level (4.3 ± 1.6) (Figure 1g, blue bars). This level corresponds to one-third of the expression level of the *Mbd1* exon 1 (13.6 ± 3.1), which results from both the upstream and endogenous *Mdb1* promoters. Finally, a primer pair targeting the last *Mbd1* exon (exon 16) displays much lower expression levels (1.6 ± 0.7), indicating that *Mbd1* transcripts might undergo transcriptional elongation arrest and/or incomplete post-transcriptional maturation (Figure 1g).

In HP1-TKO mouse liver, we observed only a slight difference in the expression levels of the ERV internal sequence (RTLR6-int) compared to control livers and no significant difference for its LTR (Figure 1f, compare orange and blue bars). In contrast, transcript levels issued from the TSS of the upstream promoter (TSS LTR) are significantly increased in HP1-TKO compared to control livers (q_up_ = 35.0 ± 2.5 vs. 4.3 ± 1.6, respectively, i.e., Δ_up_ = 8.1 fold upregulation) (Figure 1g, compare orange and blue bars). Levels of exon-1-containing transcripts are also increased in HP1-TKO compared to control livers (q_ex1_ = 76.5 ± 16.4 vs. 13.6 ± 3.1, respectively, i.e., Δ_ex1_ = 5.6-fold upregulation). Since exon-1-containing transcripts originate from both the endogenous and upstream promoters (q_ex1_ = q_reg_ + q_up_), we can deduce that the level of transcripts issued from the activity of the endogenous promoter undergoes a 4.4-fold upregulation (Δ_reg_ = (q_ex1_ − q_reg_)_TKO_/(q_ex1_ − q_reg_)_wt_). These results indicate that while transcripts issued from both the upstream and endogenous *Mbd1* promoters are upregulated in HP1-TKO compared to control mouse livers, the upregulation is about twice lower for the former (4.4-fold) than for the latter (8.1-fold). We conclude that both the endogenous and the ERV-derived upstream *Mbd1* promoters are controlled by HP1 proteins.

This result not only confirms that the RLTR6_Mm_ERV1 element constitutes an alternative promoter controlling expression from an upstream *Mbd1* TSS, but also suggests that this ERV-derived sequence may act as an HP1-regulated transcriptional enhancer for the endogenous *Mbd1* promoter.

Unfortunately, given the very short distance separating these elements (517 bp), a very high random contact level is expected between them and it would thus be very challenging to provide evidence of enhancer-specific interactions using Chromosome Conformation Capture (3C) approaches at the *Mbd1* locus.

### 3.2. HP1 Proteins Control Trim24 and Upstream ERV Sequences Expression

**Figure 2:**
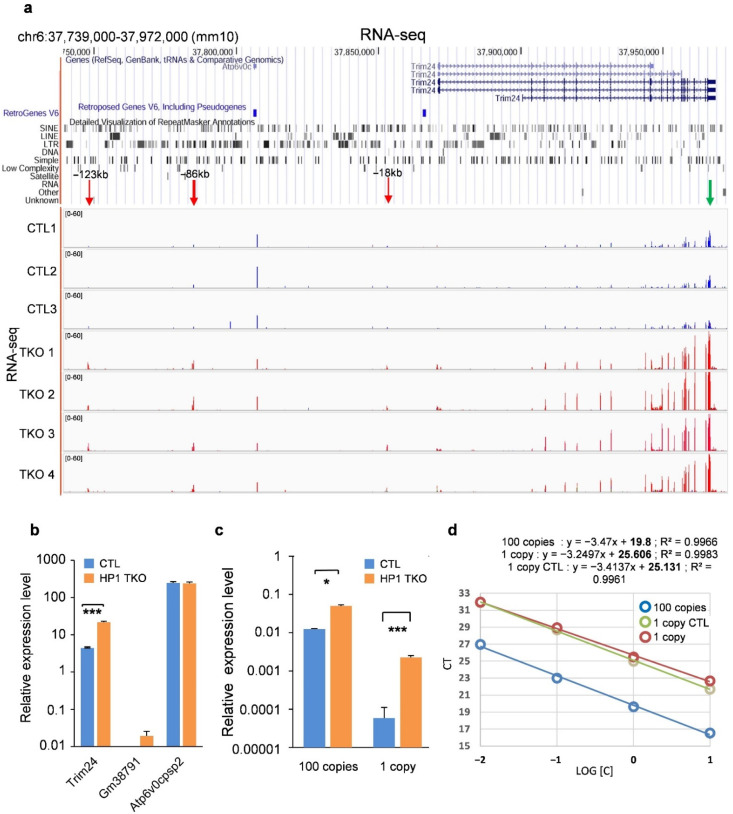
HP1 proteins control the expression of *Trim24* upstream ERV sequences. (**a**) Genome Browser snapshot of RNA-seq data at the *Trim24* locus. *Trim24* mRNA levels were found to be highly increased in the HP1-TKO compared to control livers. Upregulation was particularly clear for the last exons (green arrow on the right). One intergenic sequence, located 86 kb upstream of the *Trim24* promoter, was found to be highly de-repressed in the HP1-TKO compared to control livers (large red arrow on the left). Two other regions, located 123 kb and 18 kb upstream of the *Trim24* promoter, were also found to be de-repressed (faint red arrows on the left). (**b**) RT-qPCRs were performed on total RNA from three HP1-TKO (201, 248 and 316) and three control (202, 251 and 315) (CTL) mouse liver samples, using primers targeting *Trim24* exon 19, *Gm38791* and *Atp6v0cpsp2* transcripts. RNA levels were normalised relative to *Gapdh* mRNA levels. We observe a significant difference in expression level between the two conditions only for *Trim24*. Error bars represent the s.e.m. of three biological replicates. *p*-value < 0.01 (***), (Student’s *t*-test). (**c**) Primer pairs binding to 1 copy of RLTR6-18 ERV or 100 copies of RLTR6-86 ERV *Trim24* upstream sequences were used in RT-qPCR experiments and RNA levels were normalised relative to *Gapdh* mRNA levels. For both primer pairs, expression levels observed for the HP1-TKO was higher than in control mouse liver. Error bars represent s.e.m. of three biological replicates. *p*-value < 0.01 (***), and *p*-value < 0.05 (*) (Student *t*-tests). (**d**) Selection of primer pairs that amplify either one copy of the RLTR6-18 ERV (“1 copy”) or a hundred copies (“100 copies”) of ERV sequences in the mouse genome. The number of copies amplified by the selected primer pairs was determined on serial dilutions of genomic DNA (standard curves) in comparison with a control qPCR primer pair (“1 copy CTL”) that targets exon 1 of the *Krüppel-like factor 4* (*Klf4*) gene, which amplifies exactly one copy in the mouse genome (see Materials and Methods).

To explore the possibility that some ERV-LTRs may act as HP1-controlled long-range enhancers for endogenous genes, we chose to turn our investigations toward the *Trim24* locus where the VL30-LTR (also called RLTR6) ERV sequences are located at large distances from the *Trim24* promoter. These ERVs are particularly interesting since their expression was previously shown to be linked to the expression of *Trim24* itself and were hypothesised to behave as TRIM24-dependent enhancers [20]. We previously showed that *Trim24* is indeed upregulated in HP1-TKO livers as compared to control livers [7] (Figure 2a), and this result was again confirmed by RT-qPCR experiments, while two other genes of the locus (*Gm38791* and *Atp6v0cpsp2*) were not upregulated (Figure 2b).

Using our previously published RNA-seq data [7], we found that three ERV sequences corresponding to VL30-LTRs located 123 kb, 86 kb and 18 kb upstream of the *Trim24* promoter (RLTR6-123, RLTR6-86 and RLTR6-18, respectively) are indeed upregulated in HP1-TKO compared to control mouse livers (red arrows in Figure 2a), whereas all other transposable elements remain silent in this region (Figure 2a). This result was confirmed by RT-qPCR for the RLTR6-18 element (Figure 2c), for which a primer pair that targeted no other sequence in the entire mouse genome could be designed (Figure 2d). Unfortunately, because of the repetitive nature of ERVs, no primer pair amplifying a single copy of the two other ERV elements could be designed.

These results show that HP1 proteins control the activity of the *Trim24* promoter and of three specific upstream ERV sequences.

### 3.3. Trim24 Promoter Displays a Specific Long-Range Interaction with the RLTR6-86 ERV

**Figure 3:**
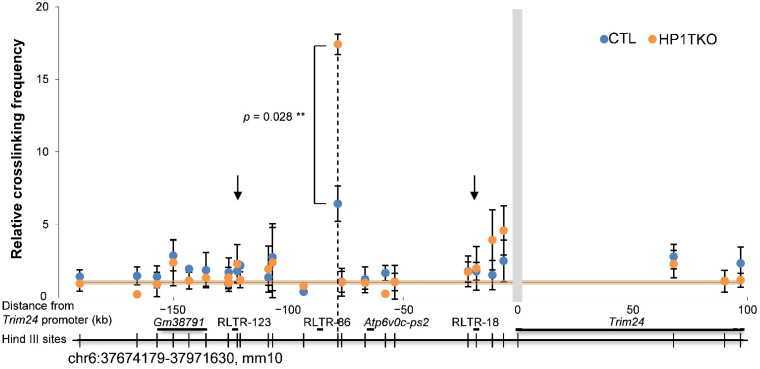
The *Trim24* promoter displays a HP1-dependent long-range interaction with the upstream RLTR-86 ERV. The 3C experiment showing the interaction between the RLTR-86 ERV and the *Trim24* promoter in control (blue dots) and HP1-TKO (orange dots) mouse liver cells. Black vertical arrows indicate experimental points corresponding to RLTR-123 and RLTR-18 ERV elements (no interactions with the *Trim24* promoter) and the vertical dashed line the RLTR-86 element. The grey vertical bar indicates the position of the HindIII fragment containing the *Trim24* promoter where the bait primer has been designed and brown horizontal bars depict the noise band of the assays (random contact level). Locations of the genes and RLTR ERVs are shown as black horizontal bars below the graph while thin vertical bars show HindIII sites used in the experiment. Error bars indicate s.e.m. of four biological replicates. *p*-value (**) (Student’s *t*-test) indicates the difference in RLTR-86/*Trim24* promoter interactions between control and HP1-TKO samples.

We then investigated the possibility that these three ERV sequences may act as transcriptional enhancers, the activities of which would be controlled by HP1 proteins. To that aim, we performed 3C-qPCR experiments using a fixed primer (bait) located in a restriction fragment containing the *Trim24* promoter. In control mouse liver (Figure 3, blue dots), we found that the *Trim24* promoter interacts much more frequently (relative crosslinking frequency of 6.42 ± 1.22) with the distant upstream RLTR6-86 ERV element (vertical dashed line in Figure 3) than with any other chromatin segment in a 300 kb surrounding area, including the RLTR6-123 and RLTR6-18 ERV sequences (vertical black arrows in Figure 3).

This result suggests that the RLTR6-86 ERV element contributes to regulate *Trim24* gene expression in wild-type mouse liver by forming a specific long-range chromatin interaction with its promoter, thus acting as a putative classical transcriptional enhancer.

### 3.4. HP1 Proteins Control Trim24 Promoter/RLTR6-86 ERV Interaction

To investigate whether HP1 proteins control this long-range interaction, we performed 3C-qPCR experiments in HP1-TKO mouse liver (Figure 3, orange dots). We found that the *Trim24* promoter/RLTR6-86 ERV-specific interaction, unlike any contact with other ERV elements in the surrounding area, is significantly increased (*p*-value = 0.028, Student’s *t*-test) in the liver of HP1-TKO mice compared to control animals (relative crosslinking frequency of 17.41 ± 0.71 vs. 6.42 ± 1.22, respectively) (Figure 3, vertical dashed line).

Altogether, these experiments demonstrate that the RLTR6-86 ERV element behaves like a putative transcriptional enhancer, forming a long-range interaction with the *Trim24* promoter, and that HP1 proteins partially prevent this interaction, thus maintaining a low expression level.

### 3.5. RLTR6-86 ERV Is Characterised by a Specific Epigenetic Landscape with Both HP1-Dependent and HP1-Independent Features

Enhancer-reporter assays performed in primary Bipotential Mouse Embryonic Liver (BMEL) cells, using the RLTR6-86 ERV and the firefly luciferase under the control of the SV40 promoter, showed that this ERV element does not possess intrinsic enhancer activity (Supplementary Figure S2). This result suggests that other determinants present in the endogenous context might be required for its putative *Trim24*-specific enhancer activity. It may thus be interesting to address this point in future studies by removing the RLTR6-86 ERV by genome-editing approaches.

In order to determine whether the putative enhancer activity of the RLTR6-86 ERV element correlates with a specific epigenetic landscape at the endogenous *Trim24* locus, we performed chromatin immunoprecipitation and qPCR (ChIP-qPCR) analyses on the livers of 8-week-old control and HP1-TKO mice.

**Figure 4:**
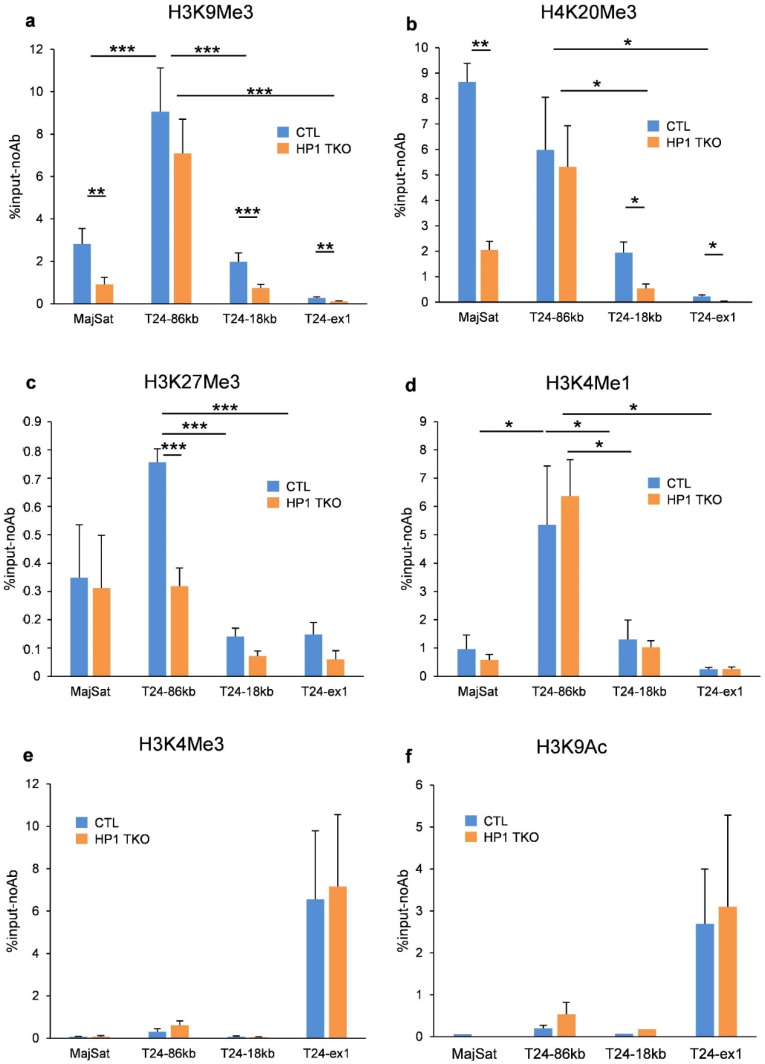
Epigenetic landscape at the *Trim24* gene locus. ChIP-qPCR experiment showing the quantification of different histone modifications in control (blue bars) and HP1-TKO (orange bars) 8-week-old mouse livers at the major satellite repeats (MajSat), at the RLTR6-86 ERV (86 kb), the RLTR6-18 ERV (18 kb) and *Trim24* exon1 (Trim24ex1). ChIP-qPCR quantifications are shown as the difference between the percentage of input with antibodies for the different histone modifications and the percentage of input in the absence of antibody (%input-NoAb). (**a**) H3K9me3, (**b**) H4K20me3, (**c**) H3K27me3, (**d**) H3K4me1, (**e**) H3K4me3 and (**f**) H3K9Ac. Error bars indicate s.e.m. of at least three biological replicates. *p*-values (Student’s *t*-test) indicate the difference in quantifications between control and HP1-TKO samples or between different genomic sites in control samples: *p*-value < 0.01 (***), *p*-value < 0.02 (**), and *p*-value < 0.05 (*).

In control animals, we found that the RLTR6-86 ERV element is specifically enriched in the two constitutive heterochromatin marks H3K9me3 and H4K20me3 (*p*-value of 0.004 and 0.039, respectively), as well as in the facultative heterochromatin mark H3K27me3 (*p*-value = 0.0004), as compared to the RLTR6-18 ERV element (Figure 4a–c). These repressive marks were barely present in the *Trim24* promoter region (Trim24ex1, Figure 4a–c).

We found that of these three marks, only H3K27me3 is significantly reduced at the RLTR6-86 ERV element in HP1-TKO compared to control animals (Figure 4c, comparing the blue and orange bars), whereas the H3K9me3 and H4K20me3 marks remain unchanged (Figure 4a,b, comparing the blue and orange bars). A similar observation can be made at the *Mbd1* LTR-TSS, although the levels of the different marks are not as high as for *Trim24*, possibly due to the proximity of the *Mbd1* promoter (Supplementary Figure S3).

Interestingly, H3K9me3, H4K20me3 and H3K27me3 are also present at the pericentromeric heterochromatin-associated major satellite repeats and at the *Trim24* RLTR6-18 ERV, but in contrast to the RLTR6-86 ERV, H3K9me3 and H4K20me3 are here significantly reduced in HP1-TKO compared to control, whereas H3K27me3 remains unchanged (Figure 4a–c, comparing the blue and orange bars). We next checked the presence of the enhancer-specific mark H3K4me1 and found that this mark is significantly enriched at the RLTR6-86 ERV element as compared to RLTR6-18 ERV in both control and HP1-TKO livers (*p*-value = 0.05 and 0.002, respectively) (Figure 4d, red and green stars, respectively). Finally, we found that the two marks associated with transcriptional activity, H3K4me3 and H3K9Ac, are present exclusively at the *Trim24* exon1 and at similar levels in control and HP1-TKO livers (Figure 4e,f).

**Figure 5:**
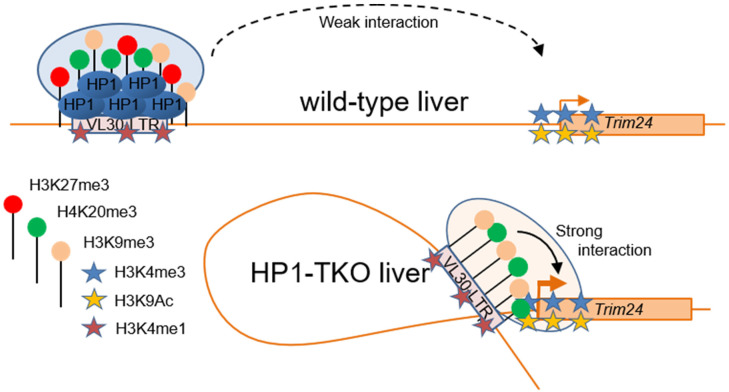
A model for HP1-dependent VL30-LTR ERV transcription control at the *Trim24* locus. In wild-type liver, the RLTR-86 VL30-LTR is covered by a repressive complex (blue shaded oval) triggered by H3K9me3, H4K20me3 and H3K27me3 repressive marks in the presence of the H3H4me1 enhancer mark. This peculiar epigenetic bookmarking prevents the long-range interaction of this ERV with the *Trim24* promoter. Upon loss of HP1 (HP1-TKO), H3K27me3 is specifically decreased leading to the release of the repressive complex and recruitment of specific transcription factors (pale orange oval), thus allowing the association of this ERV with the *Trim24* promoter and increasing the expression of this gene.

We conclude that the RLTR6-86 ERV is characterised by an enrichment of the enhancer-associated mark H3K4me1, together with a high enrichment of the heterochromatin-associated marks H3K9me3 and H4K20me3, which do not appear to interfere with its upregulation in the absence of HP1. In contrast, H3K27me3 levels on the RLTR6-86 sequence decrease upon HP1 depletion, and this decrease is associated with increased interaction frequencies with the *Trim24* promoter and the upregulation of this gene (Figure 5).

## 4. Discussion

We recently showed that the HP1 proteins are essential for preventing liver tumorigenesis in the mouse. We also showed that HP1 depletion leads to the reactivation of specific ERVs within adult livers, correlating with the transcriptional upregulation of surrounding genes [7]. In order to decipher the molecular mechanisms underlying this correlation, we investigated specific VL30-LTRs at two loci that are upregulated in HP1-TKO mouse livers, the *Mbd1* and *Trim24* gene loci, the deregulated expression of which has previously been associated with liver tumorigenesis [11,12].

At the *Mbd1* locus, we reveal that a VL30-LTR element acting as an alternative promoter also favours the activity of the endogenous *Mbd1* promoter when the HP1 proteins are depleted. This result suggests that this ERV-derived element may act as a transcriptional enhancer, the activity of which is controlled by HP1 proteins, although this hypothesis cannot be tested because of the short distance between this element and the *Mbd1* promoter, which is incompatible with 3C analyses.

At the *Trim24* locus, our 3C-qPCR experiments show that the promoter of this gene interacts physically with a specific distal VL30-LTR element in control mouse livers and that this interaction strongly increases upon HP1 depletion, correlating with *Trim24* upregulation. These results suggest that this VL30-LTR may act, as classical transcriptional enhancers do, by favouring endogenous gene expression through a direct long-range chromatin interaction with the gene promoter, and that this interaction is regulated by HP1. It is noteworthy that three genes are located within 100 kb surrounding this VL30-LTR, *Trim24*, *Gm38791* and *Atp6v0cpsp2*, amongst which only *Trim24* is upregulated in HP1-TKO mouse livers, demonstrating that the putative enhancer activity of the VL30-LTR at this locus is gene-specific. This is a very frequent situation for classical enhancers in mammals to control one or several specific genes, sometimes over hundreds of kb, but not all genes of a locus, depending on the specific determinants of both the enhancers and the associated promoters (for a review, see [21]). This result is in line with a previous observation [20] showing that, upon the loss of the corepressor TRIM24, increased recruitment of RNA Polymerase II is observed at the *Trim24* promoter, as well as at the level of several ERV-derived elements, including the HP1-dependent VL30-LTR, in correlation with their upregulation (our data and [20]). In the present study, we also confirm that H3K4me3 is present specifically at the *Trim24* promoter and, as observed upon loss of the TRIM24 protein [20], we show that it is unchanged upon the loss of HP1, indicating that the level of H3K4me3 is not directly linked to the level of *Trim24* expression. Our results also corroborate previous evidence indicating that the TRIM24 protein can interact with HP1, although the functional relevance of this interaction has not yet been demonstrated [9]. We may therefore hypothesise that HP1 could be necessary for *Trim24* activity and/or recruitment at a specific ERV.

Finally, we show that, compared to another VL30-LTR of the locus (i.e., RLTR6-18), the RLTR6-86 ERV displays a specific epigenetic landscape characterised by an enrichment in three marks known to be associated with heterochromatin [22], H3K9me3, H4K20me3 and H3K27me3, as well as a mark generally associated with enhancer identity, H3K4me1 [23]. Similar results were obtained at the *Mbd1* VL30-LTR. Since these elements behave like putative enhancers that are controlled by the HP1 proteins, it is thus perhaps not unexpected that they possess both a typical enhancer epigenetic mark (H3K4me1) and heterochromatin marks. Surprisingly, of all these enriched marks, only the facultative heterochromatin-associated mark H3K27me3 is significantly reduced in HP1-TKO compared to control livers, whereas H3K9me3 and H4K20me3 remain similarly enriched. In contrast, we observe that H3K9me3 and H4K20me3 are decreased at major satellite repeats and at the RLTR6-18 ERV in HP1-TKO mice compared to control animals. This last result was expected according to the model of HP1-dependent deposition and the maintenance of H3K9me3 and H4K20me3 at pericentromeric heterochromatin [24]. Altogether, our results demonstrate that, compared to other ERVs of the loci, the *Mbd1* VL30-LTR and *Trim24* RLTR6-86 ERVs have specific features, being enriched in H3K4me1 as well as in H3K9me3 and H4K20me3 marks even in the absence of HP1, a result reminiscent of the observation that specific genomic regions remain protected against histone eviction in sperm chromatin [25,26]. This suggests that these constitutive marks might behave here as bookmarks for ERV-derived and/or HP1-dependent enhancers. The co-occurrence of H3K9me3 with other marks, and in particular with H3K27me3, has already been observed at specific genomic loci. However, the exact role of these different marks remains quite enigmatic [27,28]. Our data suggest that H3K27me3, and most likely their associated Polycomb Group proteins, found at some VL30 LTRs, are critical for maintaining the HP1-dependent repression of the *Trim24* and *Mbd1* genes, whereas H3K9me3, H4K20me3 and H3K4me1 would participate in the identity of these VL30 LTRs as enhancer elements rather than in the regulation of their expression per se.

The role of the HP1 proteins as activating or repressing factors for endogenous gene regulation, as well as for the silencing of transposable elements, remains highly debated (for a recent review, see [29]). In this regard, relevant studies in model organisms such as *Drosophila* can help the interpretation of experiments performed in more complex genomes such as those of mammals. While tethering studies, which bring HP1 proteins to reporter genes, support a role for HP1 as repressors [30], gene expression approaches upon HP1 knockdown indicate that the impact on gene regulation is more complex with both upregulated and downregulated transcripts [31]. Remarkably, however, in both mammals [7] and *Drosophila* [32], transposable elements are clearly upregulated upon HP1 knockdown, demonstrating a repressive role of the HP1 proteins in this context. Interestingly, different mechanisms of the HP1-dependent silencing of transposable elements have been proposed in *Drosophila*, including the direct binding of HP1 to the transposable elements as in the case of the gypsy-like element, ZAM [33], or by allowing the expression of small RNAs that themselves will silence the expression of the transposable elements in the case of piRNAs clusters [34]. In mammals, different mechanisms of silencing transposable elements have also been described, the best characterised involving the corepressor TRIM28 and its interaction with HP1 [7,29]. Our work links the roles of HP1 in the control of transposable elements and endogenous gene regulation. In our model, HP1 would allow the establishment of a heterochromatin structure at specific ERVs, probably through binding with TRIM24, keeping them at large physical distance from the promoters of endogenous genes (Figure 5). Upon the withdrawal of HP1, H3K27me3 is specifically decreased in these silent ERVs, leading to their association with an open promoter characterised by high levels of H3K9ac and H3K4me3, and to an increased expression of the associated gene. Although some direct physical interactions of ERV elements with endogenous genes have been previously described [35], their quantification and functionality in the regulation of gene expression remain so far elusive. Our work constitutes, to our knowledge, the first demonstration of a direct long-range interaction between an ERV-derived sequence and the promoter of an endogenous gene in mammals, as well as the first evidence that HP1 regulates the activity of a putative transcriptional enhancer.

## Data Availability

The RNA-seq data from HP1-TKO and control mouse livers were downloaded from the GEO database (GSE119244) [7].

## Supplementary Materials

The following supporting information can be downloaded at: https://www.mdpi.com/article/10.3390/cells11152392/s1, Figure S1: HP1 gene inactivation; Figure S2: Enhancer-reporter assays in BMEL cells; Figure S3: Epigenetic landscape of the *Mbd1* gene promoter region; Table S1: Primers used for RT-qPCR, 3C-qPCR, ChIP-qPCR assays and genotyping.
