# Supplementary material for: Endogenous Retroviral Sequences Behave as Putative Enhancers Controlling Gene Expression through HP1-Regulated Long-Range Chromatin Interactions
Source: Cells. 2022 Aug 3;11(15):2392. doi: 10.3390/cells11152392 (PMC9368123; doi:10.3390/cells11152392)
Supplement: Supplementary file 1 [file cells-11-02392-s001.zip › cells-1661838-supplementary/(revised) cells-1661838 supp fig s2.pdf]

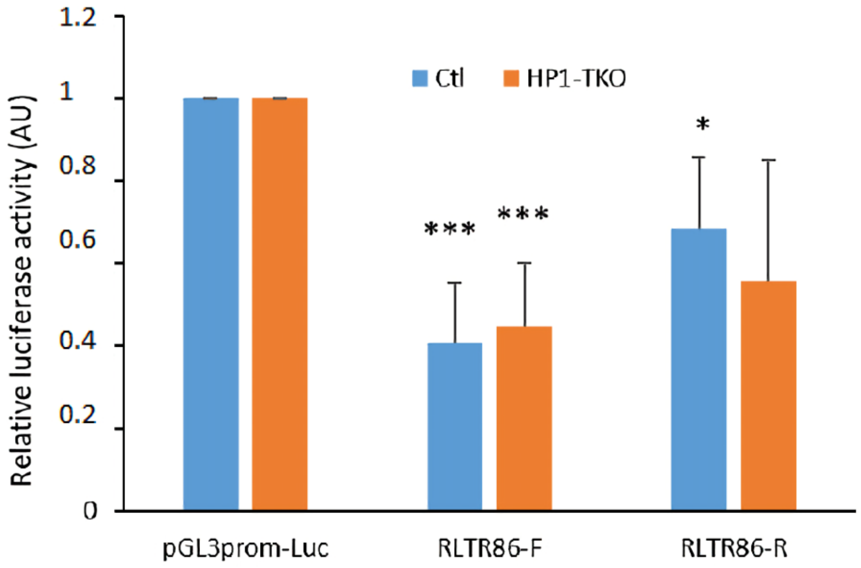

Supplementary Figure S2: Enhancer-reporter assay. The *Trim24* associated RLTR-86 ERV was cloned in forward (RLTR86-F) and reverse (RLTR86-R) orientation as compared to the endogenous *Trim24* gene. These constructs were transfected using lipofectamine in two independent control (CTL) and two independent HP1-TKO BMEL cell lines in parallel with a control vector expressing the Renilla luciferase. Error bars indicate s.e.m of at least three independent experiments. The statistical significance of the difference between each construct and the PGL3pro-Luc construct was determined by a student *t*-test. *p*-value < 0.01 (***) and *p*-value < 0.05 (*). The differences between the CTL and the HP1-TKO cells were not statistically significant for both constructs.
