# Supplementary material for: Endogenous Retroviral Sequences Behave as Putative Enhancers Controlling Gene Expression through HP1-Regulated Long-Range Chromatin Interactions
Source: Cells. 2022 Aug 3;11(15):2392. doi: 10.3390/cells11152392 (PMC9368123; doi:10.3390/cells11152392)
Supplement: Supplementary file 1 [file cells-11-02392-s001.zip › cells-1661838-supplementary/Calvet_Supp-FigS1.pdf]

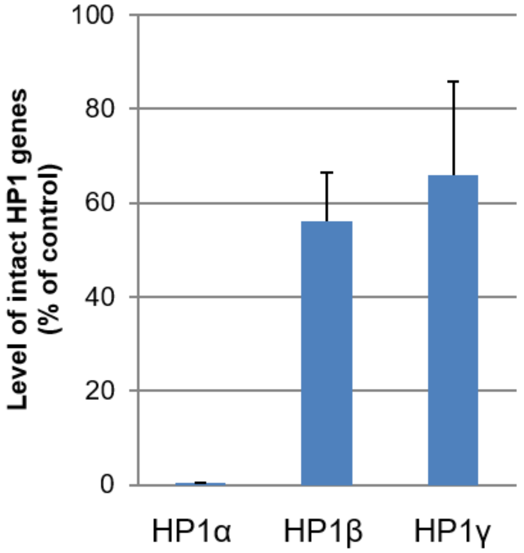

Figure S1. HP1 gene inactivation. Rates of HP1α, HP1β and HP1γ gene deletions in mouse liver have been measured on genomic DNA issued from 3C-qPCR experiments to get the best evaluation of our studied samples. Two samples were used for each genotype: 207, 316 for the HP1-TKO and 208, 315 for control mouse livers. Each mouse Control is linked to a mouse HP1-TKO by being born in the same litter. The percentage of remaining unrecombined genes was calculated as follow: (HP1TKO/CTL)*100. All data has been quantified in triplicate (error bars are s.e.m., n=2 biological replicates).
