# Supplementary material for: Endogenous Retroviral Sequences Behave as Putative Enhancers Controlling Gene Expression through HP1-Regulated Long-Range Chromatin Interactions
Source: Cells. 2022 Aug 3;11(15):2392. doi: 10.3390/cells11152392 (PMC9368123; doi:10.3390/cells11152392)
Supplement: Supplementary file 1 [file cells-11-02392-s001.zip › cells-1661838-supplementary/Calvet_Supp-FigS3_revision2.pdf]

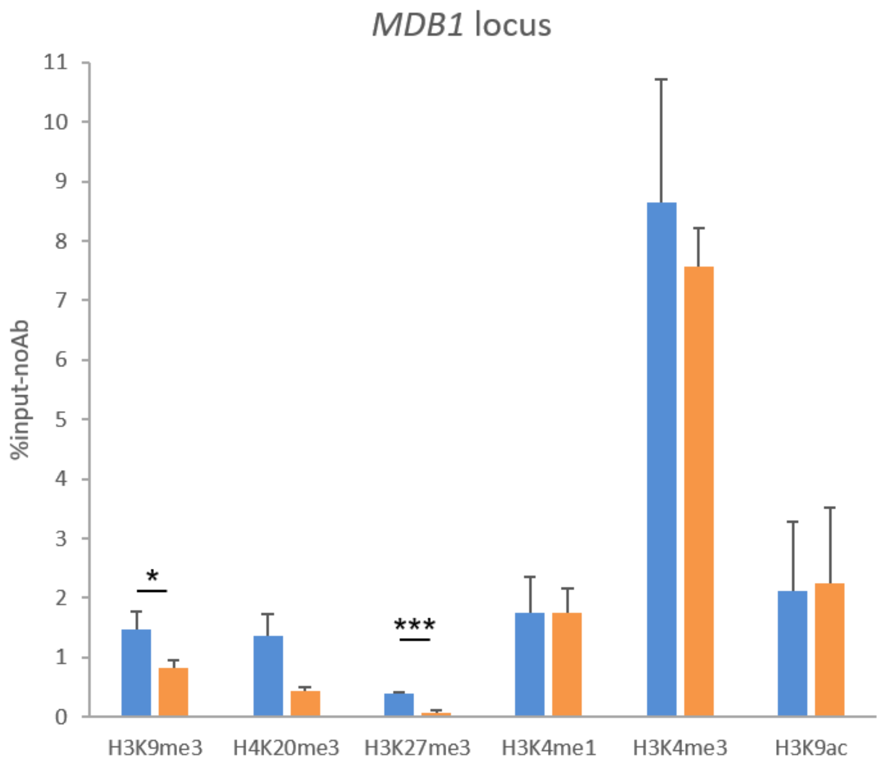

Figure S3. Epigenetic landscape of the *Mbd1* gene promoter region. ChIP-qPCR experiment showing the quantifications of different histone modifications in control (blue bars) and HP1-TKO (orange bars) 8 weeks old mouse livers at the *Mbd1* gene promoter. ChIP-qPCR quantifications are shown as the difference between the percentage of input with antibodies for the different histone modifications and the percentage of input in absence of antibody (%input-noAb). As indicated on the figure, six histone modifications have been investigated : H3K9me3, H4K20me3, H3K27me3, H3K4me1, H3K4me3 and H3K9Ac. Error bars indicate s.e.m. of at least three biological replicates. *p*-values (Student *t*-test) indicate the difference of enrichment between control and HP1-TKO samples: p-value < 0.01 (***), p-value < 0.02 (**), and p-value < 0.05 (*).
